# A novel histologic grading scheme based on poorly differentiated clusters is applicable to treated rectal cancer and is associated with established histopathological prognosticators

**DOI:** 10.1002/cam4.740

**Published:** 2016-05-11

**Authors:** Michelle Yang, Aseeb Ur Rehman, Chunlai Zuo, Christine E. Sheehan, Edward C Lee, Jingmei Lin, Zijin Zhao, Euna Choi, Hwajeong Lee

**Affiliations:** ^1^ Department of PathologyUniversity of VermontBurlingtonVermont; ^2^ Anatomic PathologyAlbany Medical CollegeAlbanyNew York; ^3^ Department of SurgeryAlbany Medical CenterAlbanyNew York; ^4^ Pathology and Laboratory MedicineIndiana UniversityIndianapolisIndiana

**Keywords:** Histologic grading, poorly differentiated clusters, rectal cancer, tumor budding, tumor regression

## Abstract

The conventional histologic grading of colorectal cancer (CRC) is less suited for resected rectal cancer following neoadjuvant chemoradiation. Enumeration of poorly differentiated clusters (PDC) is a recently proposed histologic grading scheme. We aimed to apply PDC grading to treated rectal cancer and to test the prognostic significance of this novel approach. Archived hematoxylin and eosin slides of 72 rectal adenocarcinomas resected following neoadjuvant treatment were retrieved. PDC, tumor budding, and tumor regression were assessed. The parameters were correlated with clinicopathological features and survival. PDC was strongly associated with tumor budding, perineural invasion (PNI), metastasis, and low degree of tumor regression. Tumor budding was significantly associated with lymphovascular invasion and PNI, and metastasis. Tumors with a lower degree of regression were more likely to show high pathologic T stage and advanced clinical stage. Local recurrence was associated with poor survival. PDC did not correlate with overall survival. PDC grading is applicable to resected rectal cancer status post neoadjuvant treatment and correlates with established histopathological prognosticators. PDC and tumor budding may represent a histologic spectrum reflective of the same biological significance. Validation and incorporation of these simple histologic grading schemes may strengthen the prognostic power of the histologic parameters that influence the oncologic outcome in treated rectal cancer. Further study to evaluate the significance of PDC as an oncologic prognosticator is warranted.

## Introduction

The American Joint Committee on Cancer (AJCC)/Union for International Cancer Control (UICC) classification and staging system based on tumor, node, and metastasis (TNM) is widely used to predict clinical outcome and guide therapeutic management in colorectal cancer (CRC) 1. Clinical management considers additional histologic features such as tumor grade, lymphovascular invasion (LVI) and perineural invasion (PNI), nodal micrometastasis, and tumor budding as adjunctive prognosticators to further stratify patients with CRC 2, 3, 4, 5, 6. Although conventional, the histologic grading scheme is subject to high interobserver variability 2. Variability originates from controversy regarding how to grade—overall impression versus the worst area, or purely the proportion of glands 7. In addition, the grading scheme loses its prognostic power in some histologic subtypes of CRC, such as mucinous, medullary, and micropapillary carcinomas 8, thereby limiting its utility. Microsatellite unstable (MSI‐H) CRCs may be categorized as high grade, yet are associated with a better prognosis as compared to microsatellite stable tumors 9. Furthermore, the accurate assessment of the other morphologic factors that significantly influence prognosis—LVI, nodal micrometastasis, and tumor budding—often necessitate immunohistochemical workup or multilevel sectioning, thus requiring additional resources and limiting their utility in routine clinical practice 10, 11, 12.

Recently, Ueno et al. described a novel histologic grading scheme for CRC based on poorly differentiated clusters (PDC). PDC was defined as a nongland‐forming tumor cell cluster consisting of five or more tumor cells 13. This novel grading system is relatively simple to apply with improved interobserver reproducibility compared with conventional histologic grading, and demonstrated better performance as a prognosticator of oncologic outcome compared with conventional TMN stage in stages I–III CRC 14. Moreover, when PDC grading was applied to preoperative endoscopic biopsy samples of CRC, it predicted nodal status and high pathologic TNM stage in the resection specimen, with a 78% positive predictive value 15. In pT1 CRC, PDC is a histologic predictor of nodal metastases 16.

The applicability of the PDC grading scheme has not been evaluated in rectal adenocarcinoma status post neoadjuvant therapy, a scenario where altered morphology challenges conventional assessment schemes. We aimed to determine whether PDC is applicable to treated rectal cancer, and whether it is associated with other clinicopathological variables including tumor budding, tumor regression, and survival.

## Materials and Methods

### Case selection

Seventy‐two cases of resected rectal adenocarcinoma following preoperative neoadjuvant treatment from 2002 to 2015 were randomly identified by a pathology database search. Resected rectal cancer cases with final diagnoses containing designator “y” were searched. This cohort included 44 males and 28 females, and median age of 56.9 years (range 28–85 years) at the time of surgery. Median follow‐up was 18.5 months (range <1–98.5 months). Clinical follow‐up information was obtained from the electronic medical records. Archived hematoxylin and eosin (H&E) slides were retrieved and reviewed by three pathologists. The study was Institutional Review Board approved.

### Histological evaluation

Pathologic TNM staging according to 2010 AJCC, consisting of the maximum depth of invasion (T), nodal disease (N), and distant metastases (M) was assessed. In addition, PNI, LVI, and margin status were evaluated. Conventional histologic grading was not attempted because the morphology of the tumor after neoadjuvant treatment did not conform to conventional adenocarcinoma in many cases. PDC, tumor budding, and tumor regression were evaluated as below.

- Poorly differentiated clusters (PDC). PDC was assessed using the “hot spot method” as described previously 13. In brief, H&E sections containing viable tumor were scanned at low magnification (5× objective) to identify one representative section with the most cancer cell clusters. Using a 20× objective lens in a field containing the maximum number of cancer cell clusters, the number of clusters of ≥5 cancer cells lacking gland‐like structure was counted. PDC with <5, 5–9, and ≥10 clusters were graded as G1, G2, and G3, respectively. PDC was graded regardless of the presence of mucin.
- Tumor budding. Tumor budding was defined as clusters of less than five tumor cells without definite gland formation in the invasive front of the tumor or within the tumor 17, 18, 19. Given the uneven distribution of the residual viable tumor, no distinction was made as to intratumoral versus peritumoral tumor budding. Also, tumor budding was uncommon after neoadjuvant treatment. Therefore, total number of tumor buds in a field was enumerated, when present. In brief, H&E sections of viable tumor were scanned at low magnification (5× objective) to identify an area with the most frequent budding. Using a 20× objective, the total number of clusters of <5 cancer cells (tumor buds) were counted in that area.
- Tumor regression. Modified rectal cancer regression grade (m‐RCRG) recommended by Bateman et al. 20 was further modified. First, H&E slide with the most viable residual tumor was selected at scanning magnification. Second, at low power (5× objective lens), the percentage of viable tumor was estimated in reference to the tumor bed with treatment‐induced change. Treatment‐induced change included fibrosis, edema, calcifications, inflammatory cell infiltrate, including prominent eosinophils and histiocytes, and mucin pools. The percentage of the residual tumor was divided into five categories: <5%, 5–25%, 26–50%, 51–75%, and >75%. The first group with less than 5% of residual tumor corresponded to Bateman's m‐RCRG 1, the second and third corresponded to m‐RCRG 2, and the latter two groups m‐RCRG 3. The lower the percentage of residual tumor represented a higher degree of tumor regression.
- MUC1 immunohistochemistry. Five cases were randomly selected from each PDC grade. Total 15 representative formalin‐fixed paraffin‐embedded tissue blocks were subject to immunohistochemistry against MUC1 antibody. Four‐microns thick tissue sections were incubated with the primary monoclonal antibodies against MUC1 (Clone Ma695, working dilution 1:500, Leica Biosystems, Newcastle, United Kingdom). Peripheral membranous staining of the PDC and TB toward the stroma was evaluated.

### Statistics

Pearson's chi‐square test was used to test the relationship between two parameters. Cox regression was used for survival analysis. *P* < 0.05 was considered statistically significant.

## Results

### ypTNM stage and clinical follow‐up

Pathologic TNM stage and clinical stage, and histologic parameters including LVI, PNI, and margin status are listed in Table 1. Five (7%) patients showed complete pathologic response of the tumor following neoadjuvant treatment. Eleven patients presented with biopsy proven distant metastasis. Twelve or more lymph nodes were harvested in 55 cases, with the mean of 19 lymph nodes. Less than 12 lymph nodes were harvested in 17 cases, in which 10 cases were potentially understaged due to insufficient number of lymph nodes that were all negative in the absence of metastases (clinical TNM stage I or II).

**Table 1:** Clinicopathological features of 72 patients with rectal cancer status post neoadjuvant treatment

Pathological features	No. of cases	Clinical information	No. of cases
ypTNM stage		Clinical stage	
T0	5	0	5
T1	7	I	22
T2	23	II	11
T3	33	III	23
T4	4	IV	11
N0	44	Recurrence	8
N1	21	Postop metastasis	12
N2	7	Survival at follow‐up	
M0	61	Alive	60
M1	11	Dead	6
LVI		Unknown	6
Positive	9	Gender	
Negative	63	Male	44
PNI		Female	28
Positive	8	Age	
Negative	64	<50	23
Margin		≥50	49
R0	69		
R1	3		

y, post treatment; p, pathologic; TNM, tumor, nodes, metastasis; LVI, lymphovascular invasion; PNI, perineural invasion; R0, complete resection with negative margin; R1, incomplete resection with positive margin (radial margin).

### Association between PDC versus tumor budding and tumor regression

**Figure 1:**
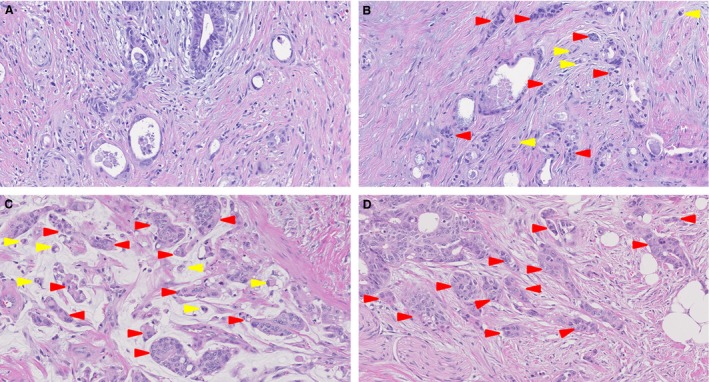
Examples of poorly differentiated clusters (PDC). (A) PDC grade 1, the tumor displays treatment‐induced changes including prominent nucleoli and eosinophilic cytoplasm in a fibrotic stroma. No PDC is noted. (B) PDC grade 2, more than five but less than 10 foci of PDC (red arrowhead) and four foci of tumor budding (yellow arrowhead) are noted within a fibrotic stroma. (C) PDC grade 3, more than 10 foci of PDC (red arrowhead) and multiple foci of tumor budding (yellow arrowhead) are noted within a pool of mucin. (D) PDC grade 3, more than 10 foci of PDC (red arrowhead) are noted within a fibrotic stroma. Hematoxylin and eosin, original magnification 200×.

Fifty‐three (74%) cases were PDC grade 1, 10 (14%) PDC grade 2, and 9 (12%) PDC grade 3 (Fig. 1).

**Figure 2:**
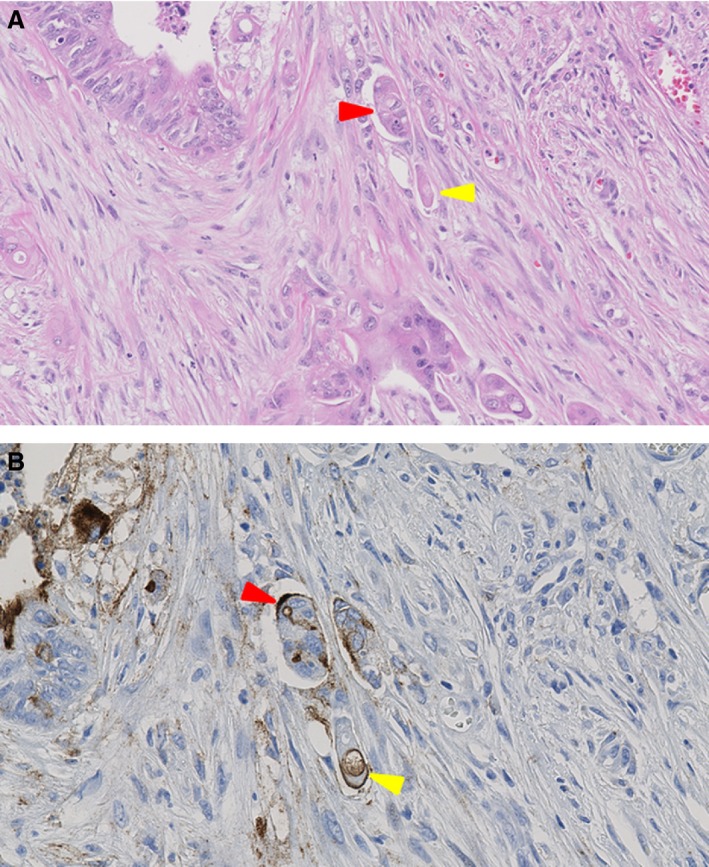
MUC1 immunohistochemical stain in poorly differentiated clusters (PDC). (A) PDC (red arrowhead) and tumor budding (yellow arrowhead) in rectal cancer following neoadjuvant treatment (hematoxylin and eosin, original magnification 200×). (B) Peripheral membranous MUC1 immunostain is focal and partial in PDC (red arrowhead) and tumor budding (yellow arrowhead) (MUC1, original magnification 400×).

Thirty‐nine (54%) showed tumor budding, of which 31 (79%) showed 1–5 tumor buds and 8 (21%) showed 6–10 tumor buds in a 20× objective lens field (Fig. 1). No case showed more than 10 tumor buds. Peripheral membranous MUC1 immunostain was focal and partial, and often negative in PDC and TB (Fig. 2).

Percentage of residual tumor of <5%, 5–25%, 25–50%, 50–75%, and >75% was observed in 17 (24%), 25 (35%), 17 (24%), 8 (11%), and 5 (7%) cases, respectively.

Pearson's chi‐square test revealed that PDC was significantly associated with tumor budding (*P* < 0.001) and tumor regression, that is, tumor with high‐grade PDC showed lower grade regression (*P* = 0.026) (Table 2). However, no significant association between tumor budding and tumor regression was observed (*P* = 0.078).

**Table 2:** Pearson's chi‐square analysis between histologic parameters versus PDC grade, tumor budding, and tumor regression

Variables	Tumor budding	PDC	Regression
ypTNM stage			
T	NS	NS	**0.015**
N	NS	NS	NS
M	**0.030**	**0.007**	NS
LVI	**<0.001**	NS	NS
PNI	**0.040**	**0.032**	**0.005**
Regression	NS	**0.026**	
Recurrence	NS	NS	NS
Postop metastasis	NS	NS	NS

Values in bold indicate statistical significance (*P* < 0.05). PDC, poorly differentiated clusters; LVI, lymphovascular invasion; PNI, perineural invasion; NS, not statistically significant (*P* ≥ 0.05).

### Association between PDC, tumor budding, and tumor regression versus ypTNM stage

PDC and tumor budding showed significant correlation with metastasis (pM) at the time of surgery. When a cutoff of 6 was applied, tumor with more than six buds tended to be of advanced clinical stage (III and IV), but statistical significance was not met (*P* = 0.058). Tumor regression showed correlation with pathologic T stage, that is, tumor with high degree of regression showed lower pT stage. Similarly, when original m‐RCRG was applied, tumors with m‐RCRG 1 and 2 showed lower clinical TNM stage than m‐RCRG 3 in TNM stages I–III tumors (*P* = 0.045).

### Survival and outcome analysis

Postoperative recurrence was associated with poor survival (*P* < 0.0001), that is, six patients had expired at the end of the follow‐up, of whom four experienced postoperative recurrence. Neither the postoperative metastases nor advanced clinical stage (III and IV) at the time of surgery showed statistically significant correlation with survival. There was no significant correlation of PDC, tumor budding, or tumor regression with recurrence, postoperative metastasis, or overall survival.

## Discussion

Prognostically relevant pathologic features such as AJCC and UICC's TNM staging, histologic type, histologic grading, the number of lymph nodes examined, lymphovascular invasion and PNI, and margin status are routinely documented in pathology reports of resected CRC. Additional histologic features such as tumor budding, tumor border, morphology suggestive of microsatellite instability, and associated precursor lesions are selectively reported as adjunctive prognosticators 21, 22, 23, 24.

For locally advanced mid or low rectal cancer, neoadjuvant radiation with or without chemotherapy is offered in order to downstage the tumor and improve the probability of complete mesorectal excision with negative margins 25, 26, 27, 28, 29. Therefore, additional pathologic features, such as the degree of tumor response to neoadjuvant treatment, degree of downstaging, radial margin status, and completeness of mesorectal envelope, have been evaluated as rectal cancer‐specific prognosticators, yet with different criteria and inconsistent results 30, 31, 32, 33, 34, 35, 36, 37.

The possibility of utilizing a novel histologic grading system that is prognostically relevant in treated rectal cancer is especially intriguing since the conventional histologic grading scheme, and subtyping is sometimes inapplicable due to altered histomorphology following treatment. For example, treated rectal adenocarcinoma displays marked cytologic atypia yet low proliferation index, and frequent neuroendocrine phenotype 38. While the degree of cytologic atypia is usually proportional to the aggressive behavior of a tumor, and neuroendocrine differentiation may portend a poor prognosis in a subset of treatment naïve colon cancer, the clinical or prognostic significance of these findings in treated rectal cancer remains unclear 38, 39, 40, 41. Likewise, large mucin pools are common following neoadjuvant treatment. By conventional subtyping, tumor with abundant mucin pools may be classified as mucinous carcinoma. Mucinous subtype in CRC might be associated with poor oncologic outcome, whereas mucin pool in treated rectal cancer may not impact prognosis 41, 42. In this study, we, for the first time to our knowledge, demonstrated that PDC is applicable to rectal cancer status post neoadjuvant treatment using the published grading criteria. PDC was also applicable to tumors with abundant mucin pools secondary to treatment. PDC grade showed positive correlation with known histopathological prognosticators including distant metastasis, PNI, and tumor regression.

Tumor budding has been extensively studied as histologic prognosticator in variable organ systems in the last decade 17, 18, 19. Despite the lack of consensus regarding the grading and methodology for assessment, tumor budding in CRC has been endorsed as a prognosticator by the UICC, European Society for Medical Oncology (ESMO), and Japanese Society for Cancer of Colon and Rectum (JSCCR) 17, 22, 43, 44.

Only a few studies evaluated tumor budding in treated rectal cancer 33, 45. Sannier et al. observed tumor budding in only 25 (22.1%) of 113 patients following neoadjuvant treatment, and tumor budding was associated with local recurrence 33. Huebner et al. reported that microscopic foci of 10 or more tumor buds—commonly used criteria for high‐grade budding in the literature 46, 47—were seen in 24 (10.1%) of 247 patients, and the presence of this degree of budding was negatively correlated with cancer‐specific survival on univariate analysis 45. In our study, tumor budding was seen in 54% of cases, and “high‐grade” budding consisting of more than 10 buds was virtually absent. Tumor budding showed positive correlation with well‐established prognosticators such as LVI, PNI, and metastasis. Tumors with less budding tended to be associated with lower TNM stage. Thus, tumor budding may also be used as an adjunctive histologic prognosticator if treated rectal cancer‐specific assessment methodology and associated thresholds are devised and verified.

Tumor budding and PDC showed strong correlation (*P* < 0.001), with the area of the most tumor budding also showing the most robust PDC. In addition, both PDC and tumor budding showed positive correlation with metastasis. Strong correlation between PDC versus tumor budding has been consistently observed in published studies including the original study of PDC 13, 15, 48. Likewise, PDC and tumor budding were positively correlated with nodal disease, metastasis, and LVI in the literature 14, 15, 16, 49, 50, 51, 52. Taken together, these observations appear to support a postulation that tumor budding and PDC likely represent a histologic spectrum of the same biologic significance, and represent the site of active mesenchymal–epithelial transition 53. More importantly, this biologic phenomenon appears to sustain after neoadjuvant therapy, therefore demonstrating greater potential as a histopathological prognosticator in treated rectal cancer. From a practical standpoint, grading of PDC was easier than enumeration of tumor budding, due to the larger size of the tumor cell clusters and simplified grading scheme of PDC in the background of fibrosis and inflammation induced by treatment.

It is noteworthy that in earlier histopathologic studies of colon cancer, the definition of budding differed from the current. Budding was defined as “microtubular cancer nests” or “undifferentiated cells” that are budding from the advancing front of the tumor. While the microtubular cancer nests were small glands, there was no cut‐off for the number of tumor cells in the “undifferentiated cells.” In retrospect, budding with undifferentiated cells in earlier studies represented a mixture of tumor budding and PDC by current definition 54. Using the earlier definition, budding was associated with lymphatic invasion, nodal disease, and decreased survival in pT3 rectal adenocarcinoma that was not radiated 55. This observation indicates that combining PDC and tumor budding may strengthen the prognostic significance.

The association between degree of tumor regression following chemoradiation and oncologic outcome is well established for rectal cancer showing complete pathologic response with no residual tumor 56, 57, 58, 59, 60. The prognostic significance of partial pathologic response has remained less clear; however, recent large scale studies of rectal adenocarcinoma demonstrated that both 5‐tier and 3‐tier regression grading systems bear prognostic significance in oncologic outcome including disease‐free survival 61, 62. We chose modified rectal cancer regression grade (m‐RCRG) in this study because of its high interobserver agreement (published kappa score 0.734), and objective criteria using percentages of residual tumor relative to tumor bed, but not the subjective descriptive terms that are commonly used in other systems 20. The degree of tumor regression was associated with PDC as well as other prognosticators including pathologic T stage, PNI, and clinical TNM stage.

The rate of complete pathologic response was 7% in our cohort, which is lower than reported range of 15–20% 60. This may be due to our approach to identify cases. We used our pathology reporting system to identify rectal cancer status post neoadjuvant treatment; that is, we searched for pathology reports of resected CRC with a designator “y” in the final diagnostic line to identify treated rectal cancer. Therefore, cases that were not staged in the pathology report due to the absence of residual tumor might have been missed. Second, the entire macroscopic tumor bed was typically submitted for microscopic examination in our cohort. Thus, rare foci of viable tumor might have been detected that would not have been detected on representative sections. Nevertheless, no adverse outcome was observed in the patients with complete pathologic response.

Cox regression analysis was attempted to correlate variable clinical and histopathologic parameters with oncologic outcomes including recurrence, postoperative metastasis, and survival. Postoperative recurrence was associated with poor survival. However, statistical significance was not met for other parameters possibly due to overall short follow‐up and low recurrence rate. Moreover, in contrast to previous report of tumor budding in treated rectal cancer, no case showed tumor budding consisting of more than 10 buds in our study 45. This indicates that low case number may be accountable for the lack of statistically significant correlation between the PDC, tumor budding, and survival in the current study. Long‐term and larger scale studies to establish the prognostic significance of PDC in reference to oncologic outcome appear warranted. Moreover, evaluation of the prognostic relevance of PDC in preneoadjuvant biopsy samples relative to postoperative outcome would be of interest. Being a tertiary referral center, pretreatment biopsy samples were not available in most of the cases.

It is possible that the PDC grading scheme may have captured treatment‐induced artifacts as well as poorly differentiated components. MUC1 immunohistochemical stain was shown to demonstrate reverse polarity—peripheral membranous staining toward the stroma—of PDC and TB in treatment‐naïve colon cancer 53. Thus, MUC1 immunostain was carried out on randomly selected cases to evaluate whether these clusters demonstrate reverse polarity reflective of poor differentiation. The staining was focal and often negative in PDC and TB, thereby limiting the assessment. However, it is noteworthy that similar observation was made in an immunohistochemical study of micropapillary carcinoma of the colon. In the study, the author showed that peripheral membranous staining for MUC1 in the PDC was minimal in several cases, and sometimes no staining was seen 63. Therefore, focal or absent peripheral membranous staining for MUC1 in the PDC and TB is not inconsistent with poor differentiation. Moreover, the positive correlation between PDC and TB versus other established histopathological prognosticators including metastasis, PNI, LVI, and tumor regression further supports the notion that these tumor clusters likely represent poorly differentiated components that sustained neoadjuvant treatment.

Additional histologic features such as stromal fibrosis, inflammation, surface ulcer, and stromal calcifications in the tumor bed were reported to be associated with oncologic outcome in treated rectal cancer 33, 40. We attempted to validate these findings. However, these features did not show significant association with PDC, tumor budding, tumor regression, or survival (unpublished data), and it was challenging to quantify these variables with confidence.

## Conclusions

PDC grading independent of gland formation is applicable to treated rectal cancer and shows positive correlation with established histopathological prognosticators. PDC and tumor budding appear to represent a histologic spectrum of the same biologic significance, which persists following neoadjuvant treatment. Further studies in a larger cohort with longer follow‐up to establish the prognostic significance of PDC in reference to oncologic outcome appear warranted.

## Conflict of Interest

None declared.
